# Isokinetic eccentric exercise can induce skeletal muscle injury within the physiologic excursion of muscle-tendon unit: a rabbit model

**DOI:** 10.1186/1749-799X-2-13

**Published:** 2007-08-21

**Authors:** Yang-Hwei Tsuang, Shui-Ling Lam, Lien-Chen Wu, Chang-Jung Chiang, Li-Ting Chen, Pei-Yu Chen, Jui-Sheng Sun, Chien-Che Wang

**Affiliations:** 1 Department of Orthopedic Surgery, Taipei City Hospital, Taipei, Taiwan; 2 Department of Physical Medicine & Rehabilitation, Cardinal Tien Hospital, Taipei, Taiwan; 3 Department of Research and Development, Healthbanks Biotechnology Corporation Ltd, Taipei, Taiwan; 4 Department of Orthopedic Surgery, National Taiwan University Hospital, Taipei, Taiwan; 5 Institute of Biomedical Engineering, National Yang-Ming University, Taipei, Taiwan; 6 Department of Orthopedic Surgery, PoJen General Hospital, Taipei, Taiwan

## Abstract

**Background and Purpose:**

Intensive eccentric exercise can cause muscle damage. We simulated an animal model of isokinetic eccentric exercise by repetitively stretching stimulated triceps surae muscle-tendon units to determine if such exercise affects the mechanical properties of the unit within its physiologic excursion.

**Methods:**

Biomechanical parameters of the muscle-tendon unit were monitored during isokinetic eccentric loading in 12 rabbits. In each animal, one limb (control group) was stretched until failure. The other limb (study group) was first subjected to isokinetic and eccentric cyclic loading at the rate of 10.0 cm/min to 112% (group I) or 120% (group II) of its initial length for 1 hour and then stretched to failure. Load-deformation curves and biomechanical parameters were compared between the study and control groups.

**Results:**

When the muscle-tendon unit received eccentric cyclic loading to 112%, changes in all biomechanical parameters – except for the slope of the load-deformation curve – were not significant. In contrast, most parameters, including the slope of the load-deformation curve, peak load, deformation at peak load, total energy absorption, and energy absorption before peak load, significantly decreased after isokinetic eccentric cyclic loading to 120%.

**Conclusion:**

We found a threshold for eccentrically induced injury of the rabbit triceps surae muscle at between 12% and 20% strain, which is within the physiologic excursion of the muscle-tendon units. Our study provided evidence that eccentric exercise may induce changes in the biomechanical properties of skeletal muscles, even within the physiologic range of the excursion of the muscle-tendon unit.

## Background

In the musculoskeletal system, muscle is the only tissue that can actively develop tension. When skeletal muscle is stimulated, it rapidly changes from passive tissue to active tissue. This change can cause muscular injuries, primarily strains or tears, which are extremely common in professional and amateur athletes [1,2]. In sports medicine, stretching exercises are often recommended to prevent injury and to improve performance [3,4]. However, intensive exercise training can result in muscular damage and soreness, especially when the exercise involves eccentric contraction [5,6].

Researchers have demonstrated that eccentric contractions create more force than either isometric or concentric contractions [7,8]. McCully and Faulkner reported that the extent of injury was related to the peak force developed during a lengthening contraction [8]. The increased development of force may be responsible for muscular injury in eccentric contraction [7]. Later, Jones et al studied the influence of mechanical factors (ie, force on long-lasting changes in voluntary force occurrence) and found that the generation of low-frequency fatigue and muscular injury is length dependent rather than force dependent [9].

To investigate the deleterious effects of eccentric exercise on humans, scientists usually use biochemical and electrophysiologic parameters to indirectly monitor the degrees of muscular injury [10-14]. An evaluation of the biomechanical properties of skeletal muscle includes an assessment for macroscopic tears. However, this method does not apply to evaluate the potential deleterious effect of eccentric exercise on the biomechanical properties of human skeletal muscles.

In a rabbit model, Lieber and Frieden demonstrated that high force per se does not cause muscular damage after eccentric contraction, but rather the magnitude of the active strain does [15]. It has been demonstrated that the triceps surae muscle-tendon unit behaved viscoelastically and the extent of muscle injuries was closely related with the stretch rate. The muscle-tendon unit tolerated great tensile force and endured high energy at fast stretch status [16]. The extent of muscular injuries were closely related to the stretch rate; with fast stretch rates, an increased peak tensile force was required, and energy absorption increased [16]. In later studies of eccentric contraction, we found that when the stimulated muscle failed, the passive muscle force was dominant and closely related to the extent of stretch [17]. In these studies, a single stretch to failure produced injury.

In previous reports on injuries induced by eccentric contraction [18,19] activation of muscle tissue was usually induced by tetanic stimulation, and this kind of disturbances could result in structural changes in the muscle-tendon unit [20]. In the present study, we used low-frequency nerve stimulation (10 Hz) to prevent the possible confounding effect of tetanic-nerve stimulation on the muscle during the experiment.

Cyclic stretching of the triceps surae muscle-tendon unit can substantially affect its tensile properties [21]. However, the effect of cyclic loading on the skeletal muscle-tendon unit during an eccentric model is still unclear. A threshold for stretch-induced injury can be reproduced at 25% strain of the triceps surae muscle-tendon unit [18]. In this study, the muscle eccentric contraction was simulated by repetitively stretching stimulated muscle-tendon units. We hypothesized that eccentric cyclic loading could produce a deleterious effect on the unit at relatively low strain level and that isokinetic eccentric exercise affected the mechanical properties of the unit, even within its physiologic excursion.

## Methods

### Animal preparation

This study was approved by the National Taiwan University Medical College's Animal Research Committee. Twelve New Zealand White rabbits (4 months old, mean weight 2.5 kg, SD 0.2 kg) were equally divided into two groups. In group I, the triceps muscle-tendon unit was passively stretched to 112% of its resting length, and in group II, it was stretched to 120%. The leg in each tested rabbit chosen to be the study or control leg was randomly assigned.

Preparation of the animals was the same as previously reported [16]. After the animals were anesthetized with ketamine 50 mg/kg given subcutaneously, an incision was made from the midcalf to the plantar surface of the foot on the lateral aspect of each hind limb. The Achilles tendon was isolated with special care to maintain the integrity of the neurovascular bundle and tendon insertion.

### Biomechanical test

During the test procedure, the sedated rabbits were put on supine position with the hip fixed in 90 degrees of flexion. To determine the in situ length of the muscle-tendon unit, a dial calipers accurate to 0.05 mm was used to measure the distance between the origin of the triceps surae at the distal femur and the insertion site at calcaneus with the knee while the ankle was flexed 90° [16]. The anesthetized rabbit was then placed in a frame attached to a testing machine (MTS Bionix 858, Minneapolis, MN, USA). The hind limbs were immobilized with K-wire transfixation through the proximal tibia. The distal tendinous insertion was freed by means of osteotomy at the calcaneal tuberosity and then clamped to the load cell of the test system. The muscle was passively extended to its original length before osteotomy. A 3-N preload was applied on the muscle, and its length was measured again [16].

Before the experiment, a skin incision was made over bilateral buttock region to expose the sciatic nerve. The nerve was isolated and clamped with a nerve stimulator (TENS SkylarkTM transcutaneous electrical nerve stimulator; Skylark Device Co., Ltd., ROC).

For the study group, the muscle-tendon unit of one hind limb was cyclically loaded for 1 hour at a rate of 10.0 cm/min to a strain amplitude of 12% or 20%. After the peak stretch amplitude was reached, stretching was discontinued, and the muscle-tendon unit returned to its initial resting length. To avoid the confounding effect of tetanic stimulation, low-frequency nerve stimulation was simultaneously applied to the sciatic nerve (pulse width 120 μsec, frequency 10 Hz, amplitude 12 mA) during cyclic loading. The magnitude of supramaximal nerve stimulation was based on our previous finding that muscle contraction was maximal under this condition [20]. After eccentric-cyclic passive stretching, the muscle was stretched without further electric stimulation at a constant rate of 10.0 cm/min until a macroscopic tear or a full division of ruptured muscle fragments occurred. For the control group, the muscle-tendon unit in the other hind limb was stretched at the same rate of 10.0 cm/min until failure.

The load and deformation required to deform the muscles were simultaneously recorded by using a personal computer and software (Testlink PCLAB Data Translation; Data Translation Inc., Marlboro, USA). All muscles were kept moist and at physiologic temperature (37°C) by irrigating them with warm normal saline. Additional anesthesia was given when needed. The rabbits were sacrificed at the completion of the study.

For each triceps surae muscle, the load and deformation of the muscle-tendon unit were recorded and plotted by using the computer. Deformation of the unit was measured when peak load was evident. Deformation was calculated as the length of the muscle at peak load minus its length before stretching. Load-deformation curves were generated, and slopes were measured at every linear portion. Energy absorption was calculated by measuring the area beneath the load-deformation curve; the area before the failure point represented the relative energy the muscle-tendon unit absorbed before it failed. A ratio of the energy absorption before peak load was measured by dividing the energy absorption before peak load with the total energy absorption during each test.

### Statistical analysis

Differences in the energy that the muscle-tendon unit absorbed before peak load and at full separation of the ruptured fragments were analyzed by using the paired t test. Because of the great individual variation in the strength of the triceps surae muscle, the paired t test was also used to evaluate differences between limbs of the rabbits in each group. The level of statistical significance was set at P < 0.05.

## Results

**Figure 1:**
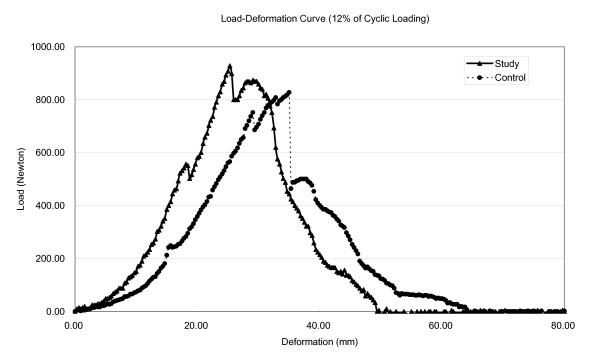
Representative load-deformation curve of a triceps surae muscle-tendon unit after isokinetic eccentric cyclic loading for 1 hour at 12% strain. The curve shows a slope of 54.9 N/mm for the study group, compared with 36.5 N/mm for the control sample.

**Figure 2:**
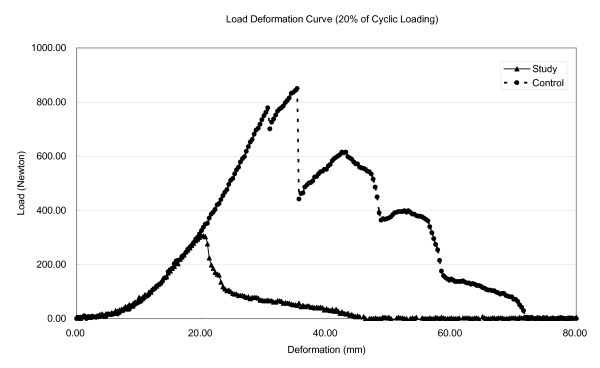
Representative load-deformation curve of the triceps surae muscle-tendon unit after isokinetic eccentric cyclic loading for 1 hour at 20% strain. All biomechanical parameters were substantially decreased in the study group. For the control and study groups, respectively, peak load was 850.5 and 305.4 N, deformation at peak load was 35.93 and 20.9 mm, the slope of the curve was 31.1 and 20.5 N/mm, total energy absorption was 23764.6 and 3989.5 N-mm, and energy absorption before peak load was 11564.5 and 2194.0 N-mm.

After isokinetic eccentric loading, all muscle-tendon units under stretch had similar curve patterns. The load-deformation curve began with an initially increasing slope and ultimately reached the peak load. After this point, a steep decline was observed, followed by a curve with gradual increasing and decreasing of the load. After 12% strain for 1 hour, the curve shows a slope of 54.9 N/mm for the study group, compared with 36.5 N/mm for the control sample. The slope of the curve was steeper in the study group than in the control group (Fig. 1). When the muscle-tendon unit was loaded to 20% strain for 1 hour, we observed a significant change on the load-deformation curve between the control and study groups. All biomechanical parameters were substantially decreased in the study group. For the control and study groups, respectively, peak load was 850.5 and 305.4 N, deformation at peak load was 35.93 and 20.9 mm, the slope of the curve was 31.1 and 20.5 N/mm, total energy absorption was 23764.6 and 3989.5 N-mm, and energy absorption before peak load was 11564.5 and 2194.0 N-mm. The peak load was lower in the study group than in the control group (Fig. 2).

**Figure 3:**
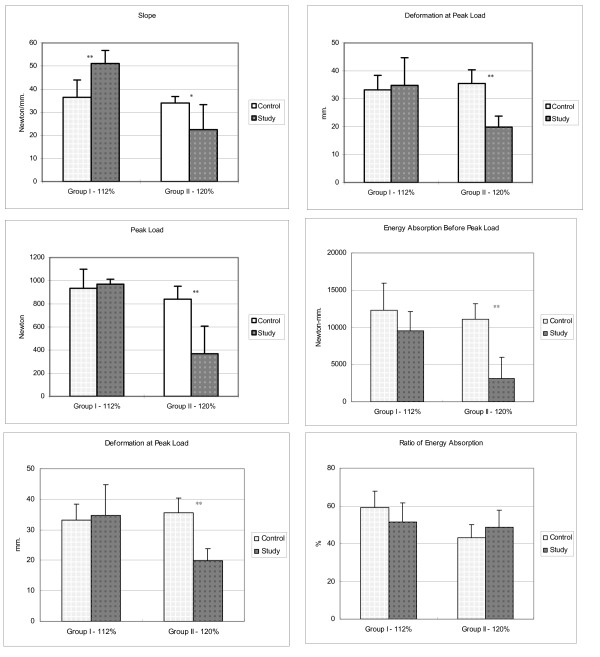
Changes in biomechanical parameters of the triceps surae muscle-tendon unit after isokinetic eccentric cyclic loading to 112% of its resting length for 1 hour. Only the slope of the load-deformation curve significantly changed. In contrast, after isokinetic eccentric cyclic loading to 120% for 1 hour, all biomechanical parameters except for the ratio of energy absorption before peak load significantly changed (*P < 0.05, **P < 0.005).

In group I (isokinetic eccentric cyclic loading to 112% of resting length), all biomechanical parameters were similar between the control and experimental limbs, except for the slope of the load-deformation curve (Fig. 3, Tables 1 &2). In group II (loading to 120% loading of resting length), all biomechanical parameters significant differed between the control and study groups, except for the ratio of energy absorption before peak load (Fig. 3, Tables 1 &2). After 1 hour of 120% loading, the slope of the load-deformation curve decreased 33.9%, the peak load decreased 57.2%, and the deformation at peak load decreased 44.0%, (Fig. 3, Table 1).

**Table 1:** Biomechanical data for triceps surae muscle-tendon units subjected to eccentric cyclic loading (n = 6)

Parameter	Group I, 112% Load	Group II, 120% Load
Slope (N/mm)		
Study	51.1 ± 5.7	22.5 ± 10.8
Control	36.5 ± 7.5	34.0 ± 2.8
P value	0.004	0.025
Peak load (N)		
Study	970.2 ± 42.1	368.6 ± 238.6
Control	934.6 ± 165. 5	840.8 ± 111.3
P value	0.327	0.002
Deformation at peak load (mm)		
Study	34.8 ± 9.9	19.9 ± 3.9
Control	33.2 ± 5.2	35.5 ± 4.9
P value	0.381	< 0.001

Note: Data other than P values are the mean (standard deviation). For all groups, the stretch rate was 10 cm/min. In the study group, stimulation was applied with 12 mA at a frequency of 10 Hz.

**Table 2:** Energy absorption of triceps surae muscle-tendon units during eccentric cyclic loading (n = 6)

	Group I, 112% Load	Group II, 120% Load
Total energy absorbed (N-mm)		
Study	18,950.0 ± 3083.8	6869.0 ± 6598.1
Control	20,740.0 ± 5380.5	25,746.0 ± 3275.0
P value	0.268	< 0.001
Energy absorbed before peak load (N-mm)		
Study	9515.4 ± 607.3	3117.7 ± 2819.3
Control	12,298.0 ± 3601.3	11,117.0 ± 2065.4
P value	0.063	< 0.001
Ratio of Energy Absorption Before Peak	Load (%)	
Study	51.4 ± 10.1	48.7 ± 9.2
Control	59.3 8.46	43.3 ± 6.7
P value	0.108	0.161

Note: Data other than P values are the mean (standard deviation). For all groups, the stretch rate was 10 cm/min. In the study group, stimulation was applied with 12 mA at a frequency of 10 Hz.

Figure 3 and Table 2 show the average total energy absorption, the energy absorption before peak load, and the ratio of energy absorption before peak load. In group I, the average total energy absorption and energy absorption before peak load remained constant. In group II, the average total energy absorption and energy absorption before peak load decreased significantly. Average total energy absorption decreased 73.3%, and energy absorption before peak load decreased 72.0% (Fig. 3, Table 2); the differences were statistically significant (both P < 0.001). No significant difference was found between the ratios of energy absorption before peak load in either groups (P > 0.05).

The sites of failure were within 0.1 to 1.0 mm from the distal musculotendinous junction for soleus muscle and within 5 to 10 mm from the distal musculotendinous junction in the lateral head of the gastrocnemius muscle. In the medial head of the gastrocnemius muscle, failure occurred within 15 to 30 mm from the distal musculotendinous junction, as previous reported [16].

## Discussion

Musculotendinous strain injuries are reportedly the most common injury in competitive athletics [1,3,22]. Their frequency and disabling effects have been documented in many epidemiologic studies [23,24]. For example, strains can cause athletes to lose time from their sport, impair their performance, and produce pain.

Eccentric contractions have been shown to produce muscle damage [25-27]. Patel et al. reported that increasing the oxidative capacity of muscle with isometric training did not protect it against eccentric contraction-induced injury [28]. The magnitude of this damage may strongly depend on the number of stretches performed, the amplitude of each stretch, and the maximum tension reached [29]. In a preliminary study, we measured excursion of the Achilles tendon between 17.8% and 22.6% strain [17]. In the present study, we investigated eccentric loading of muscle-tendon units using 12% and 20% strain under 10-Hz and 12-mA nerve stimulation to determine whether such a specific eccentric cyclic load within the physiologic range can induce muscular injury.

We previously elucidated that the loss of nerve function significantly reduced the peak force and the energy absorption before peak force [30]. The aforementioned studies were based on the tests in which specimens were loaded to rupture during a single loading test. No unloading phase was performed before rupture.

In most activities of daily living, the repetitive contraction-relaxation cycles of muscle-tendon unit are similar to dynamic cyclic loading. In this study, after isokinetic eccentric loading with 12% strain for 1 hour, the slope of the load-deformation curve was steeper in the study group than in the control group (Fig. 1). Nerve function was well preserved, and the anesthetic we used did not inhibit reflex activity [30]. We suggest that isokinetic eccentric loading with 12% strain for 1 hour can increase muscle tone of the muscle-tendon unit and thus increase the slope of load-deformation curve.

When the muscle-tendon unit was eccentrically loaded to 20% strain, we observed significant changes in the biomechanical parameters of the study group (Fig. 2). After isokinetic eccentric loading to 120% of the resting length for 1 hour, the slope of the load-deformation decreased 33.9%, the peak load decreased 57.2%, and the deformation at peak load decreased 44.0% (Fig. 3, Table 1). The average total energy absorption before the unit failed decreased 73.3%; the energy absorption before peak load decreased 72.0% (Fig. 3, Table 2). These findings suggest that eccentric contractions cause profound changes in the muscular parenchyma and that they may be the result of mechanical trauma caused by the high tension generated in relatively few active fibers during eccentric contractions [31]. Eccentric loading within the physiologic range of muscular excursion for 1 hour can induce injury of the muscle-tendon unit under this experimental condition. This observation can partially explain the mechanism of muscular injury induced by eccentric contraction during daily activities.

At a given angular velocity, the eccentric moment is greater than the corresponding concentric moment. The mode specificity of both concentric and eccentric exercises has been investigated, but the results are conflicting [32]. Eccentric activation has been well associated with delayed muscle soreness and muscle damage [31,33]. A limited number of studies have shown that isokinetic eccentric efforts may produce less muscle soreness than other exercise modalities do [31]. As a consequence, the use of this exercise modality to prevent and assess musculoskeletal injuries should be investigated further.

In 1995, Hasselman et al. studied muscular injury by using active cyclic stretching or stretching of the muscle to the point of complete muscle-tendon dissociation. They found a threshold and a continuum for active stretch-induced injury. Disruption of the muscle fibers occurred initially, and disruption of the connective tissue occurred only with large displacements of the muscle [34]. Our results are consistent with those of Kellis and Baltzopoulos. That is, eccentric activation is associated with muscular damage, even it is performed in the physiologic range [31].

Muscle strain is one of the most common injuries practicing physicians see. Until recently, little data were available on the basic science and the clinical application for the treatment and prevention of muscle strains. Certain muscles (muscles that cross several joints or those with complex architecture) are susceptible to strain injury. Commonly injured muscles include the hamstring, rectus femoris, gastrocnemius, and adductor longus muscles. All of these muscles have a strain threshold for both passive and active injury [35]. Eccentric muscle activation produces more tension in the muscle than concentric activation does, increasing susceptibility of the muscle to tearing [36]. We previously demonstrated that cyclic stretching of muscle-tendon units above a threshold drastically altered both load-deformation and failure properties [21]. Using a rabbit model in vivo, we have further demonstrated that the biomechanical parameters significantly changed after eccentric cyclic loading for 1 hour, even within physiologic range of muscular excursion (20% strain).

In summary, we demonstrated a threshold for eccentrically induced injury of the rabbit triceps surae muscle at between 12% and 20% strain, which is within the physiologic excursion of the muscle-tendon units. Our study provided evidence that eccentric exercise may induce changes in the biomechanical properties of skeletal muscles, even within the physiologic range of the excursion of the muscle-tendon unit.
